# Enhancement of Xanthate Adsorption on Cerussite Surfaces by Pb(II) Activation and Its Effect on Floatability

**DOI:** 10.3390/molecules28062455

**Published:** 2023-03-07

**Authors:** Yongchao Miao, Shuming Wen, Zhihao Shen, Qian Zhang, Qicheng Feng

**Affiliations:** 1 State Key Laboratory of Complex Nonferrous Metal Resources Clean Utilization, Faculty of Land Resource Engineering, Kunming University of Science and Technology, Kunming 650093, China; 2 Yunnan Key Laboratory of Green Separation and Enrichment of Strategic Mineral Resources, Kunming University of Science and Technology, Kunming 650093, China

**Keywords:** cerussite flotation, surface sulfidization, lead ions, xanthate adsorption

## Abstract

Cerussite is a lead oxide mineral resource that is typically enriched through sulfidization flotation. The surface sulfidation degree and the high solubility of cerussite strongly affect the flotation ability of cerussite. In the current work, lead ions were used to pretreat cerussite to intensify its sulfidization flotation. The sulfidization mechanism regulating the lead ions pretreatment on cerussite was investigated by the micro-flotation test, ToF-SIMS, zeta potential measurement, adsorption test, and XPS. The results from the micro-flotation test demonstrated that the floatability of cerussite could be improved by adding an appropriate amount of lead ions. Compared with the treatment involving only Na_2_S, the maximum recovery increased by 17.57%. Adsorption experiments showed that lead modification improved the stability of xanthate products on the surface of cerussite. According to the measurement of zeta potential and the results of ToF-SIMS, the addition of lead ion Pb pretreatment increased the number of active Pb sites adsorbed by xanthate, thereby improving the formation of hydrophobic Pb-dilute precipitate. Therefore, the interaction between lead ions and the surface of cerussite enhances the strength and stability of the hydrophobic layer, resulting in enhanced hydrophobicity of cerussite.

## 1. Introduction

As we all know, lead is a vital non-ferrous metal. Because of its good properties such as corrosion resistance, radiation protection, and ductility, lead is widely used in lead-acid batteries, aluminum sheets, aluminum alloys, metallurgy, casting, medicine, and other industries [1,2,3]; it is vital to the development of the national economy. Resources for lead minerals are plentiful, but due to modernization and the consumption of the original lead sulfide minerals, lead oxide minerals’ development is becoming increasingly crucial. Common lead oxide minerals include cerussite (PbCO_3_) and anglesite (PbSO_4_) [4,5]. The surface of lead oxide minerals is more hydrophilic and soluble; therefore, it often needs to be pre-sulfidized with sulfidizing agents such as sodium sulfide for improving the adsorption of mineral particles by xanthate-type collectors. The sulfidization flotation process has been relatively well-established and widely used in production practice [6,7,8].

In recent years, the study of cerussite has attracted increasing attention from researchers. According to Garbassi et al. [9], sulfidization of cerussite entailed the dissolution and diffusion of sulfur ions in minerals, resulting in the formation of a discontinuous porous but moderately thick PbS layer, that in turn comprises several atomic layers. According to Herrera-Urbina et al. [10], the primary reason is the difference in PbCO_3_ and PbS solubility products during the sulfidization reaction of cerussite. Feng et al. [8] found that in aqueous solutions, S in Na_2_S exists in different speciation depending on pH. For instance, below 7.0 but above 13.9, H_2_S and S^2−^ exist as the main species, respectively. When 7.0 < pH < 13.9, HS^−^ was the main chemical entity interacting with the surface of the mineral, and it was finally revealed that the HS^−^ ions generated by the moderate hydrolysis of Na_2_S under weak alkaline conditions were transferred to the cerussite surface and generated PbS species, which improves the adsorption of cerussite and xanthate. In the previous work, Feng et al. [11] investigated the influence of chloride ions on the chemistry of the cerussite pulp pretreatment solution and the characteristics of the mineral surfaces. Consequently, upon the addition of chloride ions, there were more active sites exposed on the surface of the mineral, as demonstrated by their findings. This surface makes it possible for more S species to be transported to the surface of the mineral, which is advantageous for the generation of more lead xanthate surface layers on the surface of the cerussite and an increase in the surface’s hydrophobicity. However, the research results show that in order to obtain a higher flotation recovery of cerussite, a higher concentration of Na_2_S and chloride ions should be added. This makes the efficiency of cerussite flotation greatly reduced, and it is difficult to apply to industrial production. In a recent study, Zhang et al. [12] reported the impact of Cu^2+^ on the sulfidization flotation of cerussite. It was confirmed from the results of the surface analysis that low concentrations of Cu^2+^ did not affect the xanthate adsorption but adding high concentrations of Cu^2+^ resulted in the sulfidization of cerussite. Cerussite’s surface is heavily coated with Cu and Cu-S species, which decreases the dispersion of Pb-S species and makes the subsequent flotation recovery quite difficult. Because it is difficult to control the accurate concentration of Cu^2+^ in industrial production, the addition of Cu^2+^ is difficult to carry out the flotation of cerussite. Therefore, it is necessary to study the activation flotation of cerussite and find a modification method that may be applied to industrial production.

According to earlier research, the addition of lead ions can enhance mineral flotation by increasing the collection of sulfided minerals by xanthate, promoting the sulfide flotation of oxide minerals such as azurite and malachite, and flotation in general [13,14]. In their study of lead ions-activated smithsonite, Zhang et al. [15] reported that Pb^2+^ undergoes an ion exchange reaction with the surface of smithsonite to form PbCO_3_ that can be stably adsorbed on the surface of the mineral, which has a key involvement in the sulfide flotation of smithsonite to the reinforcement. However, whether or not lead ions can activate cerussite sulfide flotation, is an area that needs to be explored further. Therefore, the addition of Pb^2+^ can have a favorable effect on the flotation of minerals.

Due to the high solubility of cerussite in the pulp system, the active sites on the mineral surface are low, and the content of free lead ions in the pulp is high, so it is difficult to carry out sulfide flotation. The purpose of this study is to add lead ions to the pulp in advance so that the newly added lead ions react with the exposed mineral surface to supplement the active sites on the mineral surface. On the other hand, the lead ions dissolved in the pulp are re-adsorbed on the mineral surface, which is beneficial to the subsequent sulfide flotation. Lead nitrate is a cheap and widely used agent, due to economic and environmental factors, and it is a suitable source of lead ions. Therefore, lead ions can be used to pretreat the mineral surface to provide favorable conditions for subsequent vulcanization. This study provides a new idea for the industrial production of cerussite and a new idea for the flotation of lead oxide ore, which has a certain reference significance. Therefore, this work is focused on investigating how Pb^2+^ affects cerussite sulfide flotation by using modern surface analysis methods and is expected to further enrich the flotation theory of cerussite.

## 2. Experimental

### 2.1. Materials

Cerussite was obtained from Guilin, Guangxi, China. After being crushed, a specimen was carefully selected and ground using an XPM-120×3 agate grinder, followed by strict sieving, and particle size adjustment to 38–74 μm for further flotation tests. The particles with a particle size below 38 μm were ground to below 2 µm using a planetary ball mill (RETSCH-MM400) for surface analysis.

**Figure 1:**
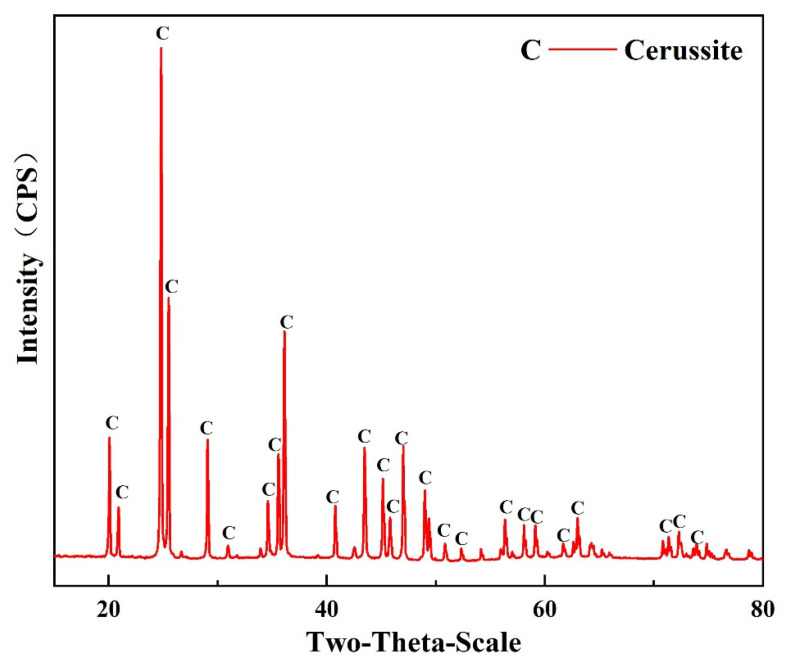
XRD pattern of the cerussite sample used in this study.

The X-ray diffraction measurement results of cerussite specimens are presented in Figure 1, showing that cerussite samples contain only a very small amount of impurities. Elemental analysis showed that the cerussite sample contains 74.3% lead, and the purity of the mineral is greater than 90%.

### 2.2. Reagents

Terpene oil was employed as a foaming agent and industrially pure sodium butyl xanthate (NaBX) was utilized as a collector in this experiment, both of which were acquired from Yunnan Raw Mineral Technology Reagent Co., Ltd.in Kunming, China. The remaining agents used in the test were analytically pure. Pb(NO₃)₂ was purchased from Tianjin Guangfu Fine Chemicals (China). For pH adjustment, 1 mmol/L H_2_SO_4_ and NaOH were used. For zeta potential measurement, 0.001 M KCl solution was used as a standard electrolyte solution. Throughout the experimental work, deionized water (prepared using the Milli Q5O system) was used. All reagents were used immediately after being prepared.

### 2.3. Micro-Flotation Tests

The micro-flotation tests were carried out at 1600 rpm in a 60 mL plexiglass container and an XFG flotation machine (supplied by the Wuhan Rock Powder Equipment Manufacturing Co., Ltd. in Wuhan, China). An amount of 2.0 g of cerussite and 40 mL of deionized water were sequentially added to the flotation cell. A certain amount of H_2_SO_4_ and NaOH were added as needed and stirred for 2 min for pH adjustments. The Pb^2+^ solution was added and then slurried for 5 min, followed by Na_2_S for sulfidization for 3 min, and then NaBX and terpene oil were added and swirled for 3 min to thoroughly engage lead ions with cerussite. Following three minutes of flotation, the foam-like product was equally scraped away by hand. The flotation concentrate and tailings products were weighed after drying for determination of flotation recovery. The mean value for each group of experiments was included in the graph after being repeated three times.

### 2.4. ToF-SIMS Analysis

When the interaction was complete, DI water was used to rinse the cerussite particles, which were subsequently dried at a constant temperature in a vacuum. Cerussite particles smaller than 37 μm were added with Na_2_S or Pb^2+^ as necessary. After drying, the materials were examined (for a 500 × 500 area) using the ToF-SIMS instrument (manufactured by the ION-ToF in Göttingen, Germany) at 96 pA and 30 keV using Bi^3+^ ions.

### 2.5. Adsorption Experiments

A total organic carbon (TOC) analyzer was used for the measurement of the quantity of adsorbed NaBX on the cerussite surface. This approach ascertains the quantity of organic matter adsorbed on the mineral’s surface via measurement of the organic carbon within the pulp and the additional TOC. A 100 mL beaker was filled with the mineral specimen (2.0 g) and DI water (50 mL), and the pulp pH and NaBX concentration were adjusted as necessary. After 30 min of stirring to attain adsorption equilibrium, the supernatant was centrifuged for 30 min before being used to calculate TOC.

### 2.6. Zeta-Potential Measurements

To obtain the zeta potential of cerussite particles under different reagent regimens, measurements were performed using a Zetasizer-3000hs, Malvern Instruments Ltd., Malvern, UK instrument. The mineral sample, already prepared for surface analysis, was then placed into a 100 mL beaker, after which 60 mL of KCl was added. Using NaOH or H_2_SO_4_, the suspension’s pH was adjusted. To obtain the same solution conditions as the micro-flotation test, other reagents were then added. A magnetic stirrer was utilized for mixing the pulp for five minutes, after which it was allowed to settle for three minutes. With the aid of a disposable pipette, the supernatant containing tiny mineral particles was removed, placed into an electrophoresis tank, and the electromotive force on the mineral surface was measured. For each pH value, three measurements were made, and the average was considered the result.

### 2.7. XPS Analysis

The preparation conditions of the samples for the XPS test were consistent with the above micro-flotation conditions. After bringing the pH to 9.5, any necessary reagents were added, and stirring was continued for 30 min. After separating the mineral samples, separating and filtering the solid and liquid components, washing multiple times with deionized water, and ultimately placing the specimens in a vacuum oven in order to achieve a constant drying temperature, a PHI 5000 VersaProbe II setup (manufactured by the ULVAC-PHI in Tokyo, Japan) equipped with an AlK-ray radiation source for ultra-high (~10^−6^ Pa) vacuum XPS was used. The obtained data were then processed by the Multi-Pak program.

## 3. Results and Discussion

### 3.1. Micro-Flotation Results

**Figure 2:**
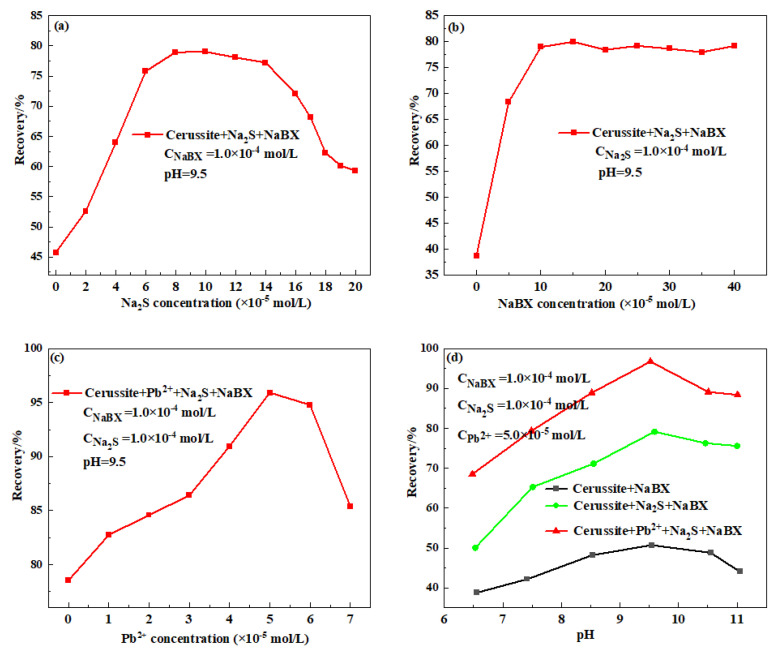
Flotation recovery of cerussite treated with different conditions: (**a**) Na_2_S concentration, (**b**) NaBX concentration, (**c**) Pb^2+^ concentration, and (**d**) pH.

Being a soluble mineral, cerussite releases a lot of Pb^2+^ into the pulp solution, which makes the mineral’s surface unstable. The significant surface polarity of cerussite is another characteristic of the mineral. The mineral becomes difficult to collect when it is introduced to the pulp because a thick and directionally densely packed hydration coating readily forms on its surface [6,8,12]. As a result, during the flotation of cerussite, its active sites must be replenished. To assess the effects of direct sulfidization and post-sulfidization with lead ions addition on the flotation performance of pure cerussite minerals, micro-flotation experiments were conducted. The flotation recovery of cerussite containing Na_2_S under 9.5 pH and 1.0 mol/L NaBX concentration conditions is depicted in Figure 2a. The generation of Pb-S species upon the surface of a mineral, whose activity was estimated by monosulfide and polysulfide matter decision, was believed to be the cause of the considerable rise in cerussite flotation recovery when Na_2_S dosage was increased from 0 to 1.0 × 10^−4^ mol/L. Additionally, it is important to note that the flotation recovery of cerussite decreases slightly when Na_2_S concentration is increased from 1.0 to 1.2 × 10^−4^ mol/L. However, as the amount of Na_2_S was increased further, it was discovered that the recovery of cerussite decreased significantly. This was because the strong negative charge created by the excess sulfur ions on the outer surface of the mineral prevented the adsorption of negatively charged xanthate on the surface of sulfide minerals. Figure 2b represents the relationship between cerussite recovery and NaBX concentration when the Na_2_S dosage is 1.0 × 10^−4^ mol/L. It is evident that the recovery of cerussite increases with the rise in NaBX concentration and continues to raise the xanthate concentration when the NaBX dosage is between 0 and 1.0 × 10^−4^ mol/L. Cerussite’s flotation recovery is typically consistent, however, the data indicate that it is only about 80%. To further enhance the recovery of cerussite, modification experiments must be conducted on it. Figure 2c displays the experimental findings on the impact of lead ions concentration on the sulfidization flotation of cerussite. This further demonstrates that a suitable quantity of the addition of lead ions can improve the flotation recovery of cerussite as the concentration of lead ions is increased from 0 to 5.0 × 10^−5^ mol/L. It is worth noting that when the lead ions concentration is greater than 5.0 × 10^−5^ mol/L, the flotation recovery decreased, which may be due to the excess lead ions added to consume Na_2_S in the flotation system, which hindered the process of cerussite sulfidization flotation. The flotation performance of cerussite as a function of pH under various reagent regimens is shown in Figure 2d. It is obvious that with NaBX alone, cerussite’s floatability is poor across the whole pH range. Due to the pulp solution’s dissolved lead ions complexing the xanthate and lowering its effective concentration, cerussite recovery was minimal. The mineral did not become hydrophobic when the lead xanthate precipitated from the bulk [16,17,18,19]. The flotation recovery of cerussite was significantly enhanced by the addition of Na_2_S. At a pH of roughly 9.5, the maximum recovery of about 80% of cerussite was attained. Additionally, it demonstrated how the flotation recovery might be aided by the addition of Na_2_S. As shown in Figure 2d, the flotation recovery of cerussite displayed a tendency to grow initially and subsequently decrease as pH increased under various reagent conditions. The optimal pH for cerussite is 9.5 since this is the pH at which the flotation recovery of cerussite is at its highest. Figure 2d also shows that when lead ions with a dosage of 5.0 × 10^−5^ mol/L are added to the flotation pulp of cerussite, and then Na_2_S and NaBX are added, the flotation recovery of cerussite is improved from 50.71% to 96.72%, further indicating that the use of an appropriate amount of lead ions is beneficial to the sulfidization flotation of cerussite.

### 3.2. ToF-SIMS Analysis

**Figure 3:**
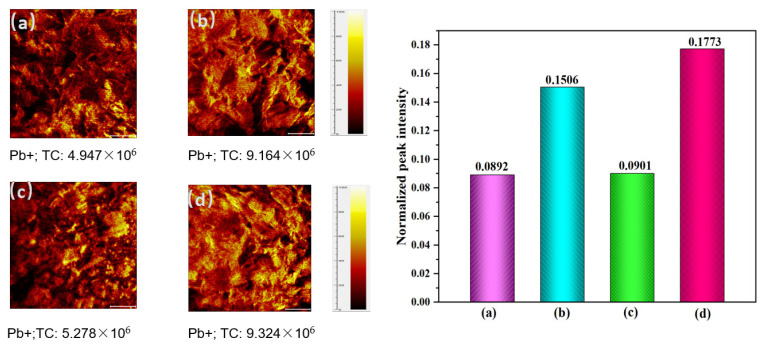
ToF-SIMS results of the 2D distribution of ions on cerussite surfaces and normalized peak intensities of sample ions treated with different conditions: (**a**) cerussite, (**b**) cerussite + Pb^2+^, (**c**) cerussite + Na_2_S, and (**d**) cerussite + Pb^2+^ + Na_2_S.

ToF-SIMS has recently become a precision instrument for mineral surface characterization and is widely used in flotation surface analysis [20]. Figure 3 shows the ToF-SIMS results of cerussite under different conditions. In the absence of Pb^2+^ and Na_2_S, positive Pb^+^ were detected on the pure cerussite (Figure 3a). Following Pb^2+^ treatment, distinctive Pb^+^ were seen in the cerussite surface’s spectrum (Figure 3b), showing that the increased amount of lead species caused the formation of more lead carbonate species. The Pb^+^ in the spectrum did not change significantly after only Na_2_S treatment (Figure 3c), but a more pronounced Pb^+^ signal appeared after treatment with Pb^2+^ and Na_2_S (Figure 3d), indicating that with the highest content of stable PbS species, the resulting PbS species are less soluble and more reactive with NaBX. Combined with the normalized intensity of secondary ions, it can be seen that the addition of Pb^2+^ increases the content of PbS, improves the activity of the cerussite surface, promotes the interaction with the collector, and thus improves the hydrophobicity of the cerussite surface. 

### 3.3. Adsorption Experiments

**Figure 4:**
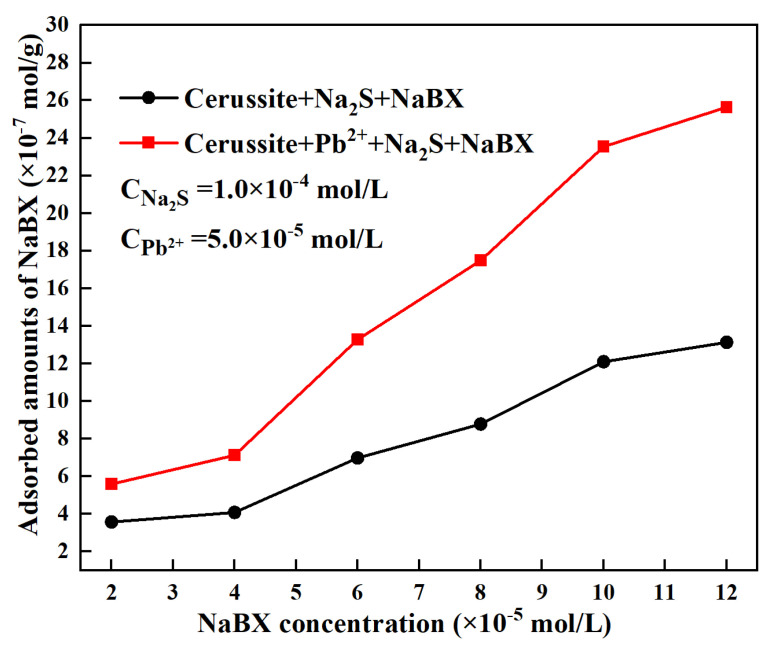
Amounts of NaBX adsorbed on cerussite surfaces as a function of NaBX concentrations before and after Pb^2+^ addition.

The behavior of the minerals during flotation is directly influenced by the adsorption of collectors upon the surface of the minerals [21]. The quantity of NaBX adsorbed upon the surface of cerussite is ascertained using the following Formula (1) once the adsorption measurement result has been obtained:

(1) $$\Gamma =\frac{\left(C\;-{\;C}_{0}\right)\times \;V}{m}$$

where C and C_0_ represent the initial and final concentration of NaBX in the solution, m refers to the weight of the mineral particles, and V stands for the volume of the solution. Figure 4 shows the calculation result, which depicts the fluctuation of NaBX adsorption on the surface of cerussite as a function of NaBX concentration in both the absence and presence of lead ions. The amounts of NaBX that is adsorbed on the cerussite surface increases with the increase in NaBX concentration whether or not lead ions are added, showing that the sulfidization of cerussite is favorable for xanthate adsorption, which corroborates quite well with the flotation results. Additionally, the amounts of NaBX that were adsorbed onto the surface of cerussite when lead ions were present and the concentration of NaBX was 1.0 × 10^−4^ mol/L was 23.54 × 10^−7^ mol/g. Only 12.09 × 10^−7^ mol/g of NaBX can adsorb on the surface of cerussite in the absence of lead ions. The adsorption of NaBX on the cerussite surface is approximately twice as high when lead ions are present compared to when lead ions are absent. According to the adsorption data, adding lead ions can improve NaBX adsorption on the cerussite’s surface.

### 3.4. Influence of Pb^2+^ on the Zeta Potential of Cerussite

**Figure 5:**
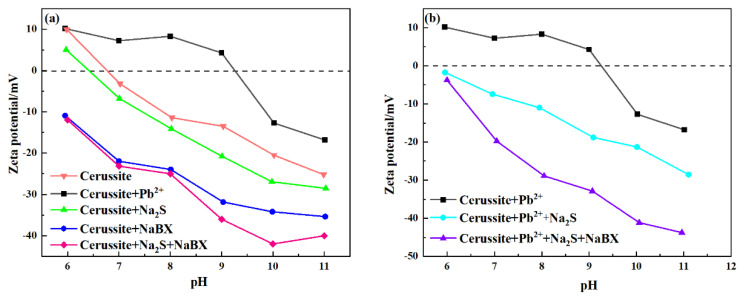
Zeta potential of (**a**) cerussite and (**b**) sulfidized cerussite as a function of pH in the presence or absence of lead ions.

We investigated the adsorption condition of flotation collectors on the material surface using the electric double-layer effect [22]. The zeta potential is a surface-sensitive technique that has been frequently used to explain flotation. It is used to look at the surface charge existing at the interface of the mineral and solution. The variations in selectivity and the presence of chemicals have an impact on flotation recovery trends [23,24]. Figure 5 depicts the zeta potential’s relationship to pH under various reagent conditions. Pure cerussite’s Isoelectric point (IEP) value in Figure 5a is around 6.5, which is similar to what has previously been described in the literature. [25]. The zeta potential of the consistent surface after Pb^2+^ treatment moved to the positive direction in the whole pH of the experiment. The flotation pulp contains lead hydroxide complexes as well as lead ions, such as Pb^2+^, Pb(OH)_4_^2−^, Pb(OH)^+^, Pb(OH)_2_, Pb(OH)_3_^−^, which are distributed as Pb^2+^ and Pb(OH)^+^ in the pH value 6–11 [26,27,28,29,30]. The selective adsorption of cationic Pb^2+^ and Pb(OH)^+^ on the cerussite surface is responsible for the positive change in the zeta potential of the cerussite surface in the addition of lead ions [31,32]. The zeta potential of cerussite altered when 1.0 × 10^−4^ mol/L Na_2_S was introduced, showing that Na_2_S reacted with positively charged lead-based molecular entities existing on the pure cerussite surface. The zeta potential of minerals following the addition of NaBX is lower than that of pure cerussite. This finding shows that a trace quantity of xanthate ions could potentially have adsorbed on the surface of the crystal. The cerussite treated with Na_2_S and NaBX had the lowest zeta potential in the measured pH range, indicating that the anionic S species in the pulp may be adsorbed on the mineral surface following Na_2_S treatment, which encourages the formation of xanthic acid in the mineral surface adsorption. The zeta potential of lead ions pretreated cerussite under various experimental settings is shown in Figure 5b. Cerussite treated with Pb^2+^ and Na_2_S had a lower zeta potential than cerussite treated with lead ions alone. The findings indicate that sulfide ions may continue to adsorb on mineral surfaces even after lead ions have been added, lowering the minerals’ surface electrical characteristics. In the Pb^2+^-Na_2_S-NaBX system, cerussite’s zeta potential shifts most quickly in the direction of negative shift, indicating that a lot of Pb^2+^ undergo interaction with the cerussite surface, thereby increasing a large number of active sites on the cerussite surface, which is advantageous to the subsequent sulfidization flotation and increases the surface hydrophobicity.

### 3.5. XPS Analysis

**Figure 6:**
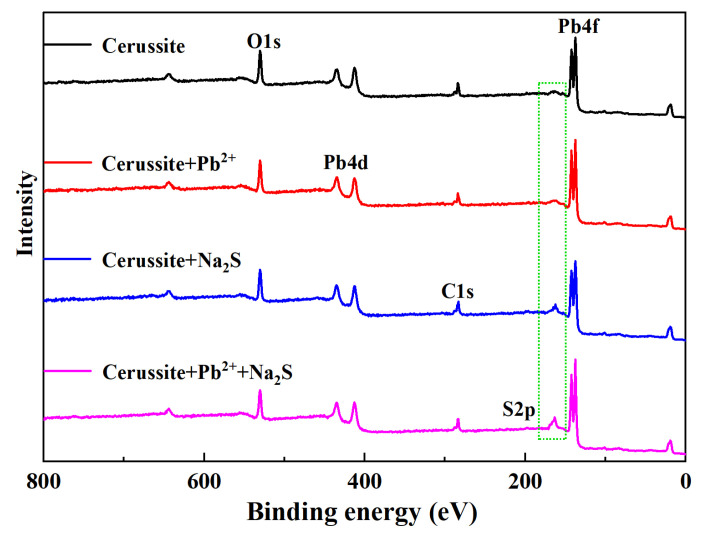
XPS survey spectra of cerussite.

XPS measures the relationship between the kinetic energy and quantity of electrons through the photoelectric effect, to judge the surface elements and chemical state of minerals [33]. XPS tests were performed to examine the elemental composition and chemical state characteristics of the mineral surfaces treated with various agents to further confirm the action mechanism of sulfidization flotation following lead ions pretreatment of cerussite [34]; Table 1 and Figure 6 display the findings. From Figure 6, it is clear that the Na_2_S-treated cerussite surface exhibits the distinctive peaks of S species. Combining with Table 1, it can be seen that after cerussite was treated with Na_2_S, the O atomic concentration dropped from 64.27% to 53.98%, the C atomic concentration decreased from 15.72% to 10.73%, while the S atomic concentration increased by 15.52%, which again supported the strong chemical reaction of Na_2_S on the cerussite surface. The O atomic concentration decreased from 64.27% to 57.68% and Pb atomic concentration increased from 20.01% to 26.14% after cerussite was treated only with Pb^2+^, showing that the Pb^2+^ were successfully adsorbed on cerussite and expanded the surface of cerussite active site. Additionally, the atomic percentage of S increased by 17.18% and the atomic concentration of Pb increased to 7.07% after the cerussite was pretreated with lead ions and then Na_2_S was added. This showed that pretreatment with lead ions makes it more likely that sulfidization reactions will occur on the surface of the cerussite.

**Figure 7:**
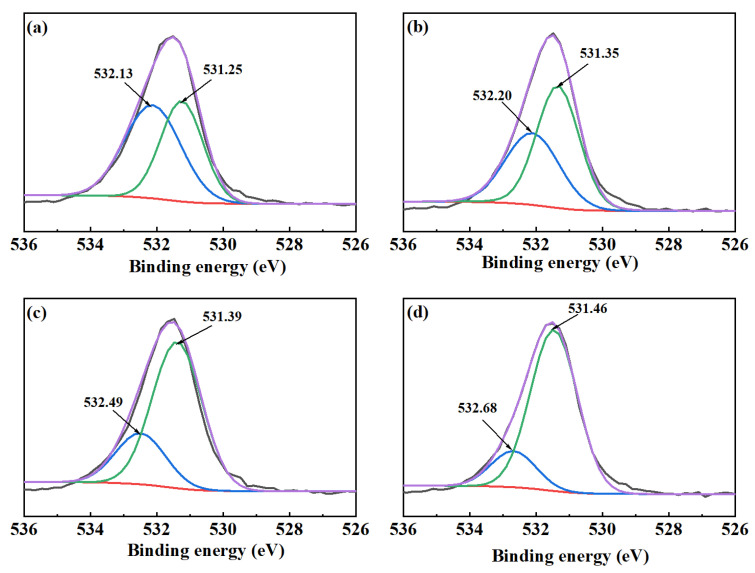
O1s XPS spectra of cerussite treated with different conditions: (**a**) cerussite, (**b**) cerussite + Pb^2+^, (**c**) cerussite + Na_2_S, and (**d**) cerussite + Pb^2+^ + Na_2_S.

**Figure 8:**
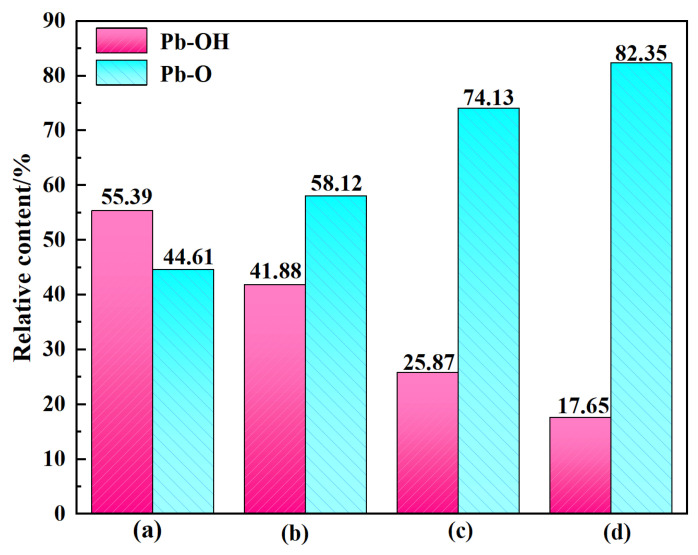
Relative atomic concentrations of O species treated with different samples: (**a**) cerussite, (**b**) cerussite + Pb^2+^, (**c**) cerussite + Na_2_S, and (**d**) cerussite + Pb^2+^ + Na_2_S.

The O1s XPS spectra of cerussite upon treatment with different conditions are displayed in Figure 7. Two spectral peaks with binding energies of 531.25 eV and 532.13 eV, respectively, can be seen in the O1s spectrum for cerussite that has undergone deionized water treatment (Figure 7a). Following lead ion treatment of cerussite, the binding energies of O1s are 531.35 eV and 532.20 eV, respectively, as shown in Figure 7b. As shown in Figure 8, the relative percentage of Pb-O on the surface of the cerussite increased by 13.51% as a result of the lead ions addition. These modifications further demonstrate that the active spots on the cerussite surface are increased by the lead ions addition. The XPS spectra of O1s after the addition of Na_2_S are seen in Figure 7c; the binding energies are 531.39 eV and 532.49 eV, respectively. The Pb-OH composition decreased by 29.52% (Figure 8), which is an outcome of the generation of more Pb-S species on the surface of the mineral, thereby weakening the hydrophilicity of the cerussite surface. This change in binding energy shows that O species have an altered chemical environment upon the addition of Na_2_S. When cerussite was treated with Pb^2+^ and Na_2_S, the binding energies of the O1s spectral peaks in Figure 7d changed to 531.46 eV and 532.68 eV, respectively. In comparison to cerussite treated only with Na_2_S, the relative content of Pb-O increased by 8.22%. This phenomenon results from the O sites on the cerussite surface reacting violently with the added lead ions, which encouraged the generation of Pb-O species. The lowest Pb-OH composition of the lead ions and Na_2_S can also be seen in Figure 8 under four different conditions. This is due to the fact that the addition of lead ions increases the number of active sites existing on the cerussite surface, leading to the formation of more sulfide layers after the action of Na_2_S, resulting in a significant decrease in the hydrophilicity of the cerussite surface, which is advantageous for the subsequent adsorption of NaBX.

**Figure 9:**
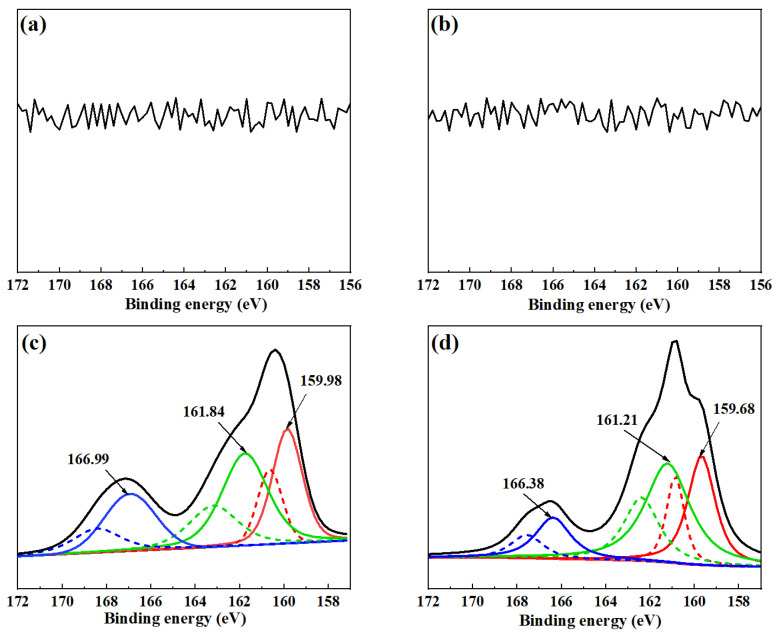
S2p XPS spectra of cerussite treated with different conditions: (**a**) cerussite, (**b**) cerussite + Pb^2+^, (**c**) cerussite + Na_2_S, and (**d**) cerussite + Pb^2+^ + Na_2_S.

**Figure 10:**
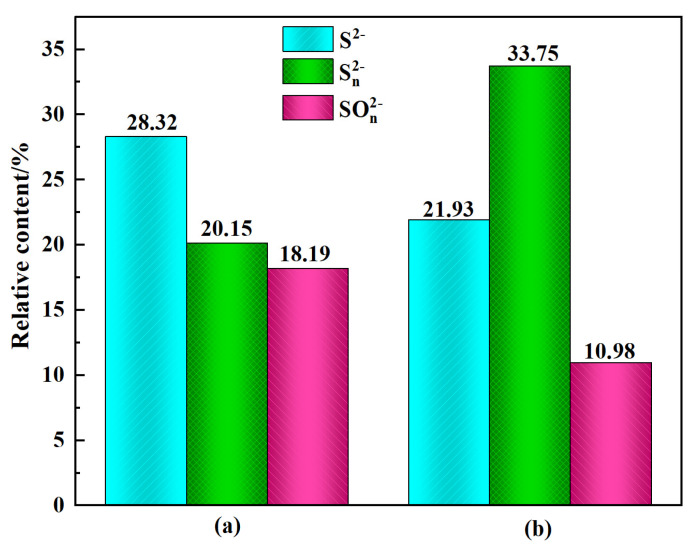
Relative atomic concentrations of S species treated with different samples: (**a**) cerussite + Na_2_S, (**b**) cerussite + Pb^2+^ + Na_2_S.

Figure 9 presents the S2p XPS spectra of cerussite surfaces treated with various reagents. The sulfided cerussite shows three pairs of S2p peaks as depicted in Figure 9c. The binding energy at 159.98 eV corresponds to the S^2−^ (monosulfide) species, the binding energy at 161.84 eV corresponds to the S_n_^2−^ (polysulfide) species, and the binding energy at 166.99 eV corresponds to the SO_n_^2−^ (sulfur oxide) species, respectively [35,36,37]. Therefore, it can be inferred that the addition of Na_2_S promotes the formation of lead monosulfide, oxysulfide, and polysulfide on the surface of cerussite. After the addition of 5.0 × 10^−5^ mol/L lead ions, the binding energies of S^2−^, S_n_^2−^, and SO_n_^2−^ species respectively shifted to 159.68 eV, 161.21 eV, and 166.38 eV, respectively (Figure 9d). The relative atomic concentration of the S^2−^ species declined from 28.32% to 21.93%, the S_n_^2−^ species grew from 20.15% to 33.75%, and the SO_n_^2−^ species decreased from 18.19% to 40.98%. (Figure 10). This implies that the S species’ chemical state has changed. The lead ions adsorbed on the surface of cerussite reacted with the added Na_2_S, and the new product increases the chemical environment of the S species on the mineral surface, as evidenced by the Pb-S^2−^ species formed on the surface of the mineral decreasing, the Pb-S_n_^2−^ species increasing significantly, and the Pb-SO_n_^2−^ species decreasing significantly. Pb and more S species undergo chemical modifications that diminish the oxidation of sulfur ions and reduce the SO_n_^2−^ component, which is advantageous for the following adsorption of xanthate ions.

## 4. Conclusions

Sulfidization is the most critical stage in the flotation of cerussite sulfide xanthate. The maximum recovery of cerussite can be increased by 17.57% by adding lead ions before sulfidation. This is because more lead sulfide species are formed on the mineral surface, which is characterized by zeta potential measurement, surface adsorption test, and XPS analysis. Lead ions pretreatment has a significant effect on the surface properties and solution chemistry of cerussite pulp solution, increasing the number of mineral surface-active centers. This surface causes the newly added lead ions in the pulp solution and the lead ions produced by the dissolution of the mineral surface to supplement the active sites on the surface of the cerussite, which is conducive to the formation of a closely connected xanthate lead surface layer on the mineral surface, thereby improving the hydrophobic mineral surface. Therefore, it is possible to improve the recovery rate of lead oxide by adding lead ions to modify the mineral surface, and it is expected to be applied to industrial production.

## Figures and Tables

**Table 1:** Relative contents on the cerussite surface under different conditions.

Samples	Element (Mass%)			
	C1s	O1s	S2p	Pb4f
cerussite	15.72	64.27	-	20.01
cerussite + Pb^2+^	16.18	57.68	-	26.14
cerussite + Na_2_S	10.73	53.98	16.52	18.77
cerussite + Pb^2+^ + Na_2_S	8.29	47.45	17.18	27.08

## Data Availability

Not Applicable.

## Sample Availability

Samples of the compounds used in the test are available from the authors.
